# Prevalence of middle ear disease in Chilean natives and the impact of development over 14 years

**DOI:** 10.1016/j.bjorl.2019.09.004

**Published:** 2019-11-03

**Authors:** Mario Tapia, Thomas Schmidt

**Affiliations:** a Complejo Asistencial Dr. Victor Ríos Ruiz, Otolaryngology Department, Los Angeles, Chile; b Universidad de Concepción, Specialty Department, Otolaryngology, Head and Neck Surgery, Concepción, Chile

**Keywords:** Chronic otitis media, Middle ear, Otitis media, Native, Quality of life

## Abstract

**Introduction:**

The prevalence of middle ear disease and its risk factors have been the subject of multiple studies. High prevalence of middle ear disease has been described among North American natives, especially chronic otitis media. Such studies have not been carried out in South America.

**Objective:**

To describe the prevalence of middle ear pathology and risk factors in native schoolchildren from southern Chile who belong to the Mapuche ethnic group, as well as the impact of socio-economic and demographic changes after 14 years of development.

**Material and methods:**

Two otologic evaluations with an interval of 14 years were performed in schoolchildren with a percentage of indigenous population above 85%. Socioeconomic and demographic data were collected from national official statistical data.

**Results:**

A total of 1067 schoolchildren were examined. Many described risk factors for ear pathology were found. An overall prevalence of 0.19% for tympanic membrane perforation, 5.6% for pars tensa retraction pockets, 1.5% for pars flaccida retraction pockets and 11.1% of otitis media with effusion was found. There were several socioeconomic improvements after 14 years. The difference between the prevalence of symptoms and the presence of otitis media with effusion was statistically significant (*p* < 0.001).

**Conclusions:**

Despite the presence of several risk factors for middle ear disease, this study population showed a low prevalence of middle ear disease. The ethnic-racial factor seems to be a protective factor.

## Introduction

The prevalence of middle ear pathology and its risk factors have induced multiple studies.^1^, ^2^, ^3^, ^4^, ^5^, ^6^, ^7^, ^8^, ^9^, ^10^, ^11^, ^12^, ^13^ Among the risk factors described the following can be found: recurrent acute otitis media, low socio economic status, low access to medical care, overcrowding, malnutrition, drinking water and sanitary facilities deficit among others. Middle ear disease rates vary according to the studied population. There are certain ethnics groups who are more likely to develop these pathologies.^14^, ^15^

The prevalence of ear pathology varies according to the country and the date of the study. In Chile, in 1973, Rosenblut et al.^16^ described a 2.12% incidence of Chronic Otitis Media (COM) in students ranging from 6 to 15 years of age living in the western area of Santiago of Chile. There was a significant difference among the socioeconomic groups, with a higher frequency in the low income group. In addition, García^17^ describe that COM was the most frequent outpatient pathology at otolaryngology units in several public clinics, which was confirmed by Wess et al.^18^ 3 years later. The last prevalence study was carried out in 1999, which described a prevalence of 0.3% for chronic otitis media in urban schoolchild population in the capital city of Chile.^19^

The high prevalence of middle ear disease and especially COM in Chile has been intuitively attributed to the miscegenation with native ethnics. This statement could be supported by the theoretical common origin with North American natives as the Apaches, Inuit and Navajos in whom higher prevalence of middle ear pathology has been shown.^14^, ^15^ However, there are no studies regarding the prevalence of middle ear disease in neither continental Chilean natives nor others among South America natives.

Indigenous communities represents 4.6% of the country population, which correspond to 692,192 citizens belonging to ethnics groups (Yámana, Rapanui, Quechua, Mapuche, Colla, Aymara, Atacameño, Alacalufe) where 604,349 are Mapuches (87.3%).^20^

Alto Biobío is a Chilean commune located in the Andes (latitude 38°37′26″ South 71°57′54″ West) that borders Argentina to the east. The annual average temperature is 11.3 °C and it has a total precipitation of 2192 mm per year. It is classified as Csb according to Köppen-Geiger, a global natural climate classification that identifies each type of climate with a series of letters that indicate the behavior of the temperatures and precipitations that characterize this type of climate.^21^ Its population is 11,486 inhabitants; 74% belong to the native Mapuche ethnic. This is a commune with high rates of rural population and low income group, as well as high rates of poor hygienic services, malnutrition, and limited access to health care services (the nearest reference hospital is 53 km away). Moreover, they suffer from a low education level, human development index and high social vulnerability. The population is exposed to the same weather, environment quality, lodging type and sanitary services. They attend the same schools and health care institutions, thus they received the same education quality, school nutrition and medical care.^22^, ^23^ School attendance is obligatory by law in Chile for 12 years. All schools establishments in this region are public.

In 2004, the opening of Ralco Hydroelectric power plant caused the displacement of the population.^24^ This event caused a significant change in the housing, sanitary and educational conditions of Alto Biobío people. It is important to mention that Alto Biobío was declared as an independent commune in 2004, and therefore the official statistics of this commune are not included with other communes only since 2006.

It was decided to focus our study on two specific objectives (1) describe the prevalence of middle ear disease in native Mapuche children and determine the impact of their ethnicity on it,and (2) analyze the results after 14 years of socioeconomic and environmental development.

Given the poor external conditions, we expected to find a high rate of middle ear pathology in the studied population. The controlled conditions in this group allowed us to avoid the predominance of extrinsic factors. Therefore, it represents a propitious group to evaluate the influence of the ethnic-racial factor on middle ear pathology.

## Methods

Two assessments were carried out in native children of Alto Biobío with 14 years of interval between them. On both occasions the evaluations were conducted in 10 schools in which more than 85% of the students belong to the Mapuche ethnic group. The sample was divided into two groups. The sample population assessed on November 2nd and 3rd 1998 was assigned as Group 1 and the sample population assessed on April 12th and 13th in 2012 was assigned as Group 2. Each evaluation was performed by three otorhinolaryngologists and residents; an otologic examination was performed using a light otoscope. The findings were classified major: tympanic membrane perforation, pars tensa retraction pockets, pars flaccida retraction pockets and Otitis Media with Effusion (OME), and minor; Dimeric tympanic membrane and myringosclerosis. The patients were asked for otologic symptoms such as hearing loss, otalgia and otorrhoea during the previous month. Socioeconomic, demographic, sanitary and educational data were collected from families of the students, school references and national official statistics.^20^, ^22^, ^23^ Collected data was analyzed by IBM SPSS v23.0.0.0 statistics program. Continuous variable (age) against dichotomous variables were analyzed with U Mann-Whitney test, Chi-square and Fisher’s test were performed for categorical dichotomous variables.

## Results

According to official national data, in 1992 a total of 234,541 Mapuches resided between the Regions of Biobío and Los Lagos of Chile, where 4656 of them lived in the Alto Biobío sector. In 2013 11,486 Mapuches were registered in Alto Biobío commune.^20^

In 1998, its rural population constituted 84.4%, the percentage of indigenous ethnicity was 71% and the average schooling was 4.98 years. They had a high rate of overcrowding with an average of 4.33 people per home (43% of households inhabited five or more people). 69.9% of homes had no access to drinking water and/or had deficient sanitary facilities and 21.88% had no equipment in their houses (electricity, gas, TV, or radio). There was only one rural health clinic for the entire commune in 1998 and they received two visits per week by general physicians from Santa Barbara (the nearest town). A total of 70% of the healthcare in this area was provided by native alternative medicine practitioners. There was no school feeding program, and 25% of children under 6 years old were underweight. In that year, the infant mortality rate was 22.73 per 1000 newborns.^20^, ^25^, ^26^

In 2012, they had a Human Development Index (HDI) of 0.637 and a Vulnerability Index (VI) of 0.674. Rural community was 80.5%, well above the regional average (16.5%). The percentage of indigenous ethnicity was 74.1% and the average level of schooling was 6.3 years. A school feeding program was already implemented in every educational establishment; 2.9% of children under 6 years were underweight. The overcrowding rate declined significantly from year to year constituting only 1.2% of households, however, 49.1% lived in poor conditions and 30.6% of the inhabitants belong to the poorest social class population (indigents). 67.7% of homes had no access to drinking water or had deficient sanitary facilities, being the commune with the lowest rate in the region.

The access to medical care is one of the factors that substantially improve health. The natives relied on 8 rural health clinics and one family health center, therefore, the people who preferred native alternative medicine has decreased significantly and now the healthcare is provided mostly by physicians. In that year, infant mortality amounted to 9.4 per 1000 newborns^20^, ^22^, ^23^ (Table 1). In both groups exposure to passive tobacco was similar and low due to the lack of access in the commune to these products and the low economic resources to maintain this habit, however, in family households the use of wood and coal stoves, without a system of ventilation is greater than the present in the urban population of the region, and therefore its exposure to biomass smoke is greater. The study subjects were evaluated in their educational establishments; other factors such as maternal nutrition, family history or daycare assistance were not analyzed.

**Table 1:** Socioeconomic and demographic data of Biobío Region in 2012, Alto Biobío commune in 1998 and 2012.

	Alto Biobío 1998	Alto Biobío 2012	Biobío Region 2012
Rural community	84.4%	80.5%	16.5%
Indigenous	71.0%	74.1%	5.22%
Schooling (years)	4.98	6.3	9.9
Poverty	NA	49.1%	21%
Indigence	21.9%	30.6%	5.2%
Underweight population in children under 6 years old	25%	2.9%	2.5%
Overcrowding	23.8%	1.2 %	0.8%
Sanitary facilities and/or drinking water deficit	69.9%	67.7%	9.2%
Native health care provider	70%	10%	<1%
Infant total mortality index	2006: 22.73/1.000 NB.	9.4/1000 NB	8.5/1000 NB

NA, Not Available data; Schooling, Average number of years of formal education in persons aged 25 and over; NB, Newborns; Overcrowding, People living in a house/number of bedrooms in the house >2.5.

A total of 1067 schoolchildren (2.134 ears) were examined, 530 students on the first assessment and 537 on the second. 47.3% were male and 52.7% female, with an average age of 9.54 years (Table 2). When performing a general homogeneity analysis between both groups, the difference of both overall averages was statistically significant. There was an overall prevalence of 0.18% for COM (2 evaluated ears), 5.6% for pars tensa retraction pockets (60 evaluated ears), 1.5% for pars flaccida retraction pockets (16 evaluated ears) and 11.1% of OME (118 evaluated ears). Minor findings were dimeric tympanic membrane in 2.53% (27 ears) and myringosclerosis in 12.9% (138 ears).

**Table 2:** General demographic results.

	1998	2012	Total
Schoolchildren examined (n)	530	537	1067
Age range (years)	3–17	0‒18	0‒18
Average age (years)	10	9.08	9.54
Gender male/female (%)	46.8/53.2	47.8/52.1	47.3/52.7
Month (season)	November (Spring)	April (Autumn)	

**Figure 1:**
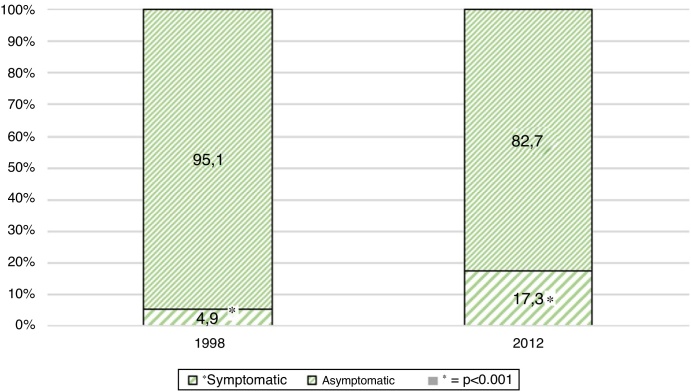
Overall prevalence of symptoms in 1998 study population (Group 1) and 2012 study population (Group 2). *Statistically significant difference between both evaluations (*p* <  0.001).

**Figure 2:**
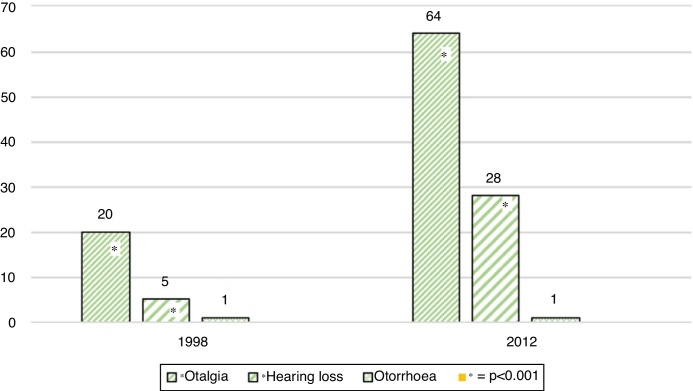
Otologic symptoms in Group 1 (1998) and Group 2 (2012). *Statistically significant difference between both evaluations (*p* <  0.001).

**Figure 3:**
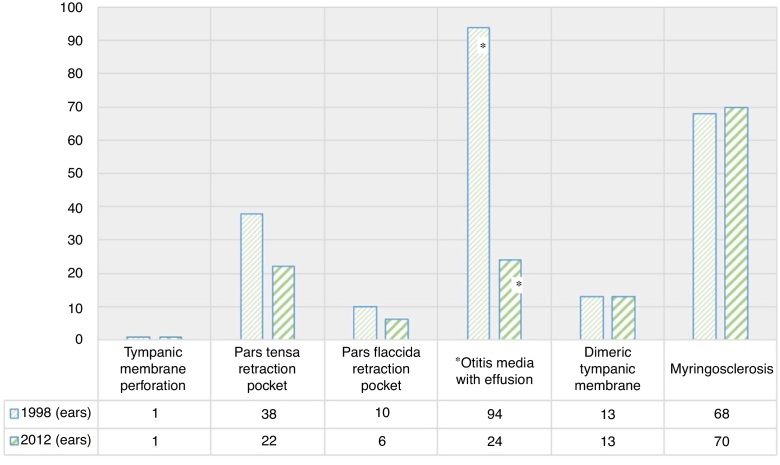
Prevalence of otologic findings per ear in 1998 and 2012. *Statistically significant difference between both evaluations (*p* <  0.001).

In Group 1 (1.998), 530 schoolchildren were evaluated. The average age was 10 years, ranging from 3 to 17 years. 53.2% were female and 46.8% were male. 95.1% were asymptomatic and 4.9% symptomatic. The symptoms were otalgia (3.8%), followed by hearing loss (0.9%) and otorrhoea (0.19%). There was a 21.1% incidence of pathologic otoscopic findings, and 13.4% of them were major findings; tympanic membrane perforation 0.18% (1 ear), pars tensa retraction pocket 3.5% (38 ears), pars flaccida retraction pocket 0.9% (10 ears) and otitis media with effusion 8.8% (94 ears) Minor findings were observed in 7.6%: dimeric tympanic membrane 1.2% (13 ears) and myringosclerosis 6.4% (68 ears) (Figure 1, Figure 2, Figure 3).

In Group 2 (2012), 537 schoolchildren were evaluated. The average age was 9.08 years, ranging from 3 to 18 years. 52.1% were female and 47.9% were male. 82.7% were asymptomatic and 17.3% symptomatic. The symptoms were otalgia (11.9%), followed by hearing loss (5.2%) and otorrhoea (0.19%). There were 12.7% of pathologic otoscopic findings, and 4.9% of them were major findings: tympanic membrane perforation 0.18% (1 ear), pars tensa retraction pocket 2.02% (22 ears), pars flaccida retraction pocket 0.55% (6 ears) and otitis media with effusion 2.23% (24 ears). Minor findings were observed in 7.8%: dimeric tympanic membrane 1.3% (13 ears) and myringosclerosis 6.5% (70 ears) (Figure 1, Figure 2, Figure 3).

**Figure 4:**
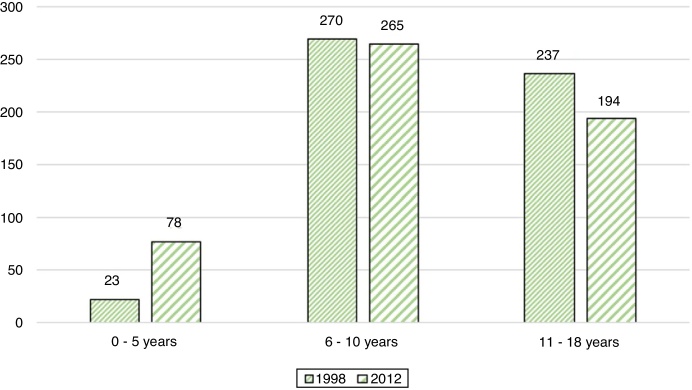
Distribution of patients in groups by age in 1998 and 2012.

The distribution of patients in each group is evaluated according to their age (Fig. 4). A homogeneity test is evaluated by Chi-square test in which the group between 6 and 10 years is homogeneous (*p* < 0.05).

Of all the evaluated findings, a statistically significant difference in symptoms and otoscopic findings were found (*p* < 0.001). Regarding symptoms, they increased from an overall 4.9% to 17.3% due to the increase of otalgia and hearing loss (*p* < 0.001). Regarding otoscopic findings, OME decreased significantly, observing a decrease from 8.8% to 2.2% (*p* < 0.05). All other parameters measured showed no significant differences.

## Discussion

The prevalence of middle ear pathology and its risk factors have been widely studied. Aspects such as gender, nutritional state, housing environmental and occupational conditions are among these factors. It has also been found that other aspects such as atopic conditions, allergic rhinitis, high respiratory tract infections, smoking, breast feeding, pharyngeal reflux, and the ethnic and racial factor have an influence on middle ear pathologies.^1^, ^2^, ^3^, ^4^, ^5^, ^6^, ^7^, ^8^, ^9^, ^10^, ^11^, ^12^, ^13^, ^14^, ^15^ The influence of these factors in middle ear pathology has been previously published with diverse results. Likewise, a higher prevalence of middle ear disease in Inuit and Eskimos has been described,^14^, ^15^, ^27^, ^28^, ^29^, ^30^ indigenous American^31^, ^32^, ^33^ and Hispanic children.^34^ The relevance of this factor in middle ear pathology has been previously proven in the literature.^35^

The population of Alto Biobío is homogeneous with respect to its socioeconomic status; there are no great socio-economic differences among the families of the community. All the schools in this commune correspond to free public education establishments, therefore the sample of the children evaluated are representative of the Alto Biobío community. We expected to find a higher prevalence of middle ear pathologies in our study. They belong to a native ethnic group exposed to several risk factors such as a high rate of poverty and indigence, adverse environmental conditions, deficient hygienic and drinking water services, and limited access to health clinics. However, our study showed that Mapuche school children from Alto Biobío had a low prevalence of middle ear pathology, similar to the Rapanui population studied by Goycoolea et al.,^36^ and lower than the study from Santiago students in 1999,^19^ Rosenblut et al.^16^ with the study in Santiago of Chile in different socioeconomic status schools, and the rate published by García^17^ multi focused study from Tarapacá region to the Magallanes region.

There have been several changes after 14 years such as the implementation of a school feeding program, mandatory schooling, better housing with a significant decrease of overcrowding and better access to medical care. However, this had no significant impact on the middle ear pathology prevalence by comparing both groups underlining a COM similar rate. Regarding the otic symptoms, the significant increase of otalgia and hearing loss presented in Group 2 is remarkable. The decreased rate of alterations observed by the otoscopic examination of Group 2 is also important, with a statistically significant decrease in otitis media with effusion (OME) cases. Due to the different season of the evaluations between both groups (spring and autumn), it is not possible to dismiss that the statistically difference of OME rates between those groups can be secondary to this external factor, thus on this point it is not possible to state a significant impact result of the socioeconomic, housing and sanitary conditions changes after 14 years.

According to indigenous genetic history of America, the indigenous population of South America could belong to Amerindians, who belong to the first people that migrated from Asia to America through Beringia.^37^ However, there was a second migration called the Nadené group, to which Apaches and Navajos belong. They have higher middle ear disease rates among the different American natives and might have a closer bond to the Eskimos who have also shown higher rates of middle ear disease. As it is showed in our study, Mapuches have a lower prevalence of middle ear disease, not similar to North American natives. It is interesting to observe the similar rates of middle ear disease in our group with Rapanui population studied by Goycoolea et al.^36^

## Conclusions

Mapuche school students from Alto Biobío have a lower prevalence of middle ear pathology than the Chilean urban continental average and North American natives. The exposure to several risk factors such as deficient environmental, socioeconomic, nutritional and sanitary conditions generates no significant impact on their low prevalence of middle ear disease. The ethnic factor seems to be an important protective factor for this population. Our findings should encourage further studies of middle ear risk factors.

## Disclosure statement

Ethical Approval: This article does not contain any studies with animals or clinical trials with humans.

Presentation information: Results were presented at the 88th Annual Meeting 2017 of the German Society of Oto-Rhino-Laryngology, Head and Neck Surgery, Congress Center der Messe Erfurt, Thüringen, Germany. May 24th–May 27th, 2017.

## Conflicts of interest

The authors declare no conflicts of interest.
